# Znf202 Affects High Density Lipoprotein Cholesterol Levels and Promotes Hepatosteatosis in Hyperlipidemic Mice

**DOI:** 10.1371/journal.pone.0057492

**Published:** 2013-02-28

**Authors:** Carlos L. J. Vrins, Ruud Out, Peter van Santbrink, André van der Zee, Tokameh Mahmoudi, Martine Groenendijk, Louis M. Havekes, Theo J. C. van Berkel, Ko Willems van Dijk, Erik A. L. Biessen

**Affiliations:** 1 Department of Medical Biochemistry, Academic Medical Center, Amsterdam, The Netherlands; 2 Division of Biopharmaceutics, Leiden/Amsterdam Center for Drug Research, Leiden University, The Netherlands; 3 Department of Human Genetics, Leiden University Medical Center, Leiden, The Netherlands; 4 Department of Biochemistry, Erasmus University Medical Centre, Rotterdam, The Netherlands; 5 TNO Prevention and Health, Gaubius Laboratory, Leiden, The Netherlands; 6 Department of General Internal Medicine, Leiden University Medical Center, Leiden, The Netherlands; 7 Department of Pathology, Maastricht University Medical Center, Maastricht, The Netherlands; UAE University, Faculty of Medicine & Health Sciences, United Arab Emirates

## Abstract

**Background:**

The zinc finger protein Znf202 is a transcriptional suppressor of lipid related genes and has been linked to hypoalphalipoproteinemia. A functional role of Znf202 in lipid metabolism *in vivo* still remains to be established.

**Methodology and Principal Findings:**

We generated mouse Znf202 expression vectors, the functionality of which was established in several *in vitro* systems. Next, effects of adenoviral *znf202* overexpression *in vivo* were determined in normo- as well as hyperlipidemic mouse models. *Znf202* overexpression in mouse hepatoma cells mhAT3F2 resulted in downregulation of members of the *Apoe/c1/c2* and *Apoa1/c3/a4* gene cluster. The repressive activity of Znf202 was firmly confirmed in an apoE reporter assay and Znf202 responsive elements within the ApoE promoter were identified. Adenoviral Znf202 transfer to *Ldlr−/−* mice resulted in downregulation of *apoe*, *apoc1*, *apoa1*, and *apoc3* within 24 h after gene transfer. Interestingly, key genes in bile flux (*abcg5/8* and *bsep)* and in bile acid synthesis (*cyp7a1*) were also downregulated. At 5 days post-infection, the expression of the aforementioned genes was normalized, but mice had developed severe hepatosteatosis accompanied by hypercholesterolemia and hypoalphalipoproteinemia. A much milder phenotype was observed in wildtype mice after 5 days of hepatic Znf202 overexpression. Interestingly and similar to *Ldl−/−* mice, HDL-cholesterol levels in wildtype mice were lowered after hepatic Znf202 overexpression.

**Conclusion/Significance:**

Znf202 overexpression *in vivo* reveals an important role of this transcriptional regulator in liver lipid homeostasis, while firmly establishing the proposed key role in the control of HDL levels.

## Introduction

Hypoalphalipoproteinemia is characterized by subnormal levels of serum high density lipoprotein (HDL) irrespective of other lipoprotein or lipid levels. It is a common lipoprotein abnormality in patients with coronary heart disease and low HDL levels were shown in various studies to be linked to risk factors like diabetes and hyperlipidemia [1], [2]. By acting as a lipid acceptor in the reverse cholesterol transport and by its anti-inflammatory properties, HDL is considered to be atheroprotective [3]–[5]. However, a causal link between genetically decreased HDL levels and an increased risk in coronary heart disease has not been established yet and the therapeutic potential of HDL is still to be determined [6]–[10].

The zinc finger protein 202 (*Znf202)* gene has been identified in the chromosomal region 11q23 that is linked to heritable hypoalphalipoproteinemia in Utah pedigrees and distinct from an apolipoprotein gene cluster ApoAI/CIII/AIV/AV [11], [12]. In contrast, a recent publication failed to confirm a correlation between genetic variations in Znf202 and HDL levels [13]. However, a sequence variation in the promoter of Znf202 did predict atherosclerosis and Ischemic Heart Disease [14]. Znf202 is a Cys_2_-His_2_ zinc-finger protein family member containing a leucine-rich SCAN domain and a Krueppel-associated box (KRAB) [15], [16]. Although the specific function of these domains is still under investigation, the presence of a KRAB domain in Znf202 is suggestive of transcriptional repressor activity [17]. This is corroborated by *in vitro* findings showing repressional activity of Znf202 on a range of target genes involved in lipid metabolism including ATP-binding cassette (ABC) transporters *Abca1, Abcg1 and* apolipoproteins *Apoe and Apoa4,* in hepatoma cells [12] as well as monocytes [18]. It was shown that Znf202 interacts with GnT response motifs in the respective promoter regions of these genes. Despite the detailed insights *in vitro*, very little is known of Znf202 mediated gene regulation *in vivo*. Moreover, the contribution of Znf202 to the regulation of serum lipids and lipoprotein metabolism is up till now unclear. But even with the contrasting findings in epidemiology studies, its activity pattern in vitro fuels the notion that Znf202 plays an important role in lipid homeostasis and is a potential candidate for a targeted therapy in cardiovascular diseases.

Considering the repressive nature of the transcription factor and, more specifically, its negative effect on the expression levels of HDL-related genes, the observed heritable low HDL cholesterol levels in those aforementioned Utah families are most likely the result of elevated activity of Znf202. Hence, we have investigated ZNF202 overexpression *in vivo* in both normo- (C57Bl/6J) as well as in hyperlipidemic (low density lipoprotein receptor knockout; *Ldlr−/−*) mice. To this end, we have generated an adenovirus vector carrying the mouse *Znf202* gene. After in vitro analysis for its repressive activity, we assessed effects of hepatic *Znf202* overexpression on serum lipid and lipoprotein levels as well as on the hepatic gene expression profile. Along with the suppressive effect on HDL cholesterol levels observed upon Znf202 overexpression in mice, these data clearly demonstrate that the transcription factor Znf202 can act as a key regulator in lipid metabolism.

## Materials and Methods

### Cell Culture

Mouse hepatoma mhAT3F2 cells [19] were cultured in Dulbecco’s Modified Eagle’s Medium (DMEM) with F-12 (Gibco) supplemented with 10% fetal calf serum (Gibco), 100 IU/ml Penicillin, 100 µg/ml Streptomycin, 20 mM GlutaMAX I, 400 nM Insulin (Sigma), 10 nM Dexamethasone (Sigma). Per.C6 and 911 cells [20], [21] were both cultured in DMEM (Gibco), supplemented with 10% fetal calf serum (Gibco), 100 IU/ml Penicillin, 100 µg/ml Streptomycin, 2 mM GlutaMAX I (Gibco), at which medium for Per.C6 cells was also supplemented with 10 mM MgCl_2_. For large scale production of recombinant adenovirus in Per.C6 cells the 10% fetal calf serum was substituted with 2% horse serum (Gibco).

### Construction of Expression and Reporter Gene Constructs

Mouse Znf202 was obtained from liver cDNA of C57Bl/6JIco by PCR (nucleotide positions 186 to 2208, GenBank accession number AF292648), with forward primer 5′ -GGTACCATAACACCCAAGAGCCAGGA -3′ containing a KpnI site and a reverse primer 5′ -TCTAGACAGAACCCATCCGTCTCAGT -3′ containing a XbaI site. The amplicon Znf202 was cloned into pShuttleCMV and pAdTrackCMV via these restriction sites [22]. The ApoE promoter (GenBank accession number D00466) was cloned from cosmid mAPOE/B1 cos27 (C2RB) by PCR. Four different promoter constructs were generated based on the absence or presence of GnT motifs, and forward primers were chosen accordingly and elongated at the 5′ with a Kpn1 restriction site to facilitate cloning. The promoter sequences were amplified by PCR with a common exon-1 targeted reverse primer carrying a 5′ Hind3 site (table S1) and amplicons cloned into the pCR.2.1 TOPO vector of the TOPO TA Cloning kit (Invitrogen). Subsequently, KpnI-HindIII fragments were cut out of the pCR.2.1 vector and inserted into KpnI-HindIII restricted pGL3-Basic (Promega). All sequences were verified by sequencing (LGTC, Leiden).

### Luciferase Reporter Assays

Transient transfections in mhAT3F2 cells were performed in 6-well plates using Fugene 6 (Roche Molecular Biochemicals). Luciferase activity of 200 ng transfected promoter-reporter constructs, with promoterless pGL3-basic serving as control, was measured by co-transfecting the cells with 100 ng of pCMV-LacZ. After 24 h, the cells were lysed with reporter lysis buffer (Promega) and luciferase activity was determined according manufacturer’s protocol (Promega) in a monolight luminometer (BD Biosciences). β-galactosidase was measured using the β-Galactosidase Enzyme Assay System in reporter lysis buffer (Promega). The effect of Znf202 overexpression on transcription regulation of the reporter gene constructs was determined by co-transfecting mhAT3F2 cells with 100 ng of a reporter gene construct and 1900 ng of the expression vector pShuttleCMV-Znf202 or an empty pShuttleCMV control vector. Luciferase activities were measured as described above and normalized for protein concentrations using BCA System (Pierce).

### Whole Cell Extract Preparation and Electrophoretic Mobility Shift Assays

911 cells were transfected with 5 µg pAdTrackCMV-Znf202 or control plasmid on 10 cm dishes using LipofectAMINE plus kit. After 40 hours cells were harvested, whole cell extract were obtained, and protein concentrations were determined by a Bradford assay (BioRad). Double-stranded [γ-^32^P]-labeled DNA probes containing the Znf202 binding sites, the consensus GnT oligonucleotide, the putative −564 and −678 Znf202 binding oligonucleotides, as well as a control unrelated fragment containing the pleiohomeotic (PHO) consensus binding site were prepared. The DNA-binding activity of mouse Znf202 in whole cell extracts to these probes was studied by means of electrophoretic mobility shift assay (EMSA). A detailed description of the EMSA protocol is provided in the Supplementary Materials and Methods (Text S1).

### Generation of Adenoviral Constructs

Recombinant adenoviral plasmids by homologous recombination of pShuttleCMV-Znf202 with pAdEasy1 were generated in BJ5183 cells (Stratagene) as described by He et al. [22]. Ad.Znf202 was produced in Per.C6 cells (Crucell) and after purification via CsCl centrifugation, the yield was assessed via a plaque assay in 911 cells. A detailed description of these techniques is provided in supplementary Materials and Methods (Text S1). The construction of control virus Ad.mock (Ad-LacZ) has been described previously [23].

### Animals

All animal work was approved by the Ethics Committee for Animal Experiments of the Leiden University (approval ID: ADEC 03054) and the experimental protocols complied with the national guidelines for use of experimental animals. Male C57Bl/6JIco and *Ldlr−/−* mice on a C57Bl/6JIco genetic background were given a standard mouse diet Chow (Hope Farms, Netherlands) and housed in conventional cages with free access to water and food.

### Treatment with Recombinant Adenovirus

*In vitro* – Murine hAT3F2 hepatoma cells were seeded in a 12-wells plate and grown to 70% confluency. Cells were infected with either Ad.Znf202 or control Ad-mock (MOI = 100). After 24 hours the cells were harvested and total RNA was isolated. *In vivo* - Recombinant adenovirus, 2×10^9^ pfu in 200 µl of PBS, was administered by injection into the tail vein of mice at the age of 28–32 weeks. Blood samples were taken via tail bleeding as indicated in the legends. After 24 hrs or 5 days post-infection, mice were sacrificed, liver sections were removed, snap-frozen in liquid nitrogen and stored at −80°C. Liver cryosections were stained with Oil Red-O (Sigma Diagnostics) and hematoxylin (Sigma Diagnostics).

### Determination of mRNA Levels

Total RNA was isolated from treated AT3F2 and liver samples using Trizol according to the manufacturer’s protocol (Invitrogen). Purified RNA was treated with RQ1 RNase-free DNase (Promega, 1 units/2 µg of total RNA) and reverse transcribed with SuperScript II Reverse Transcriptase (Invitrogen) according to the protocols supplied by the manufacturers. Quantitative gene expression analysis was performed on an ABI prism7700 Sequence Detection System using SYBR green (Applied Biosystems). PCR primers (table S2) were designed on the basis of Primer Express software with the manufacturer’s default settings (Applied Biosystems) and validated for identical efficiencies. Cyclophilin, hypoxanthine-guanine phosphoribosyl transferase (HPRT) and acidic ribosomal phosphoprotein P0 (36B4) were used as control genes.

### Lipid and Lipoprotein Analysis

Blood was collected from each individual mouse before (day 0; baseline samples) and at day 1 or at day 4 and 5 after adenovirus injection through tail bleeding in diethyl-p-nitro phenyl phosphate (paraoxon) coated capillary tubes, after a 4-hour fasting period [24]. Total plasma cholesterol (Boehringer-Mannheim) and triglycerides (Sigma Chemical Co) were measured enzymatically. From pooled plasma samples per treatment group, obtained at day 4, lipoprotein distribution was determined by fast performance liquid chromatography (FPLC). A volume of 70 µl was injected onto a Superose6 column (3.2×30 mm, AKTA-system, Pharmacia). Elution fractions of 50 µl were collected and assayed for cholesterol and triglyceride levels as described above. For measurement of liver lipids, frozen (N_2_) liver samples were homogenized and protein content was determined by a Lowry assay using BSA as calibration standard. Lipids were extracted from the homogenate according to Bligh and Dyer. After evaporation, lipids were dissolved in a 2% Triton-X-100 solution and cholesterol and triglyceride levels were determined as described above.

### Data Analysis

All results are presented as means ± SD. The significance of differences in relative gene expression was calculated using a two-tailed Student’s *t* test. Differences in lipid, lipoprotein and enzyme activity levels were analyzed statistically by Mann-Whitney-U test. Probability values less than 0.05 were considered significant.

## Results

### Znf202 Repressed the Expression of Apolipoprotein Genes in vitro

**Figure 1:**
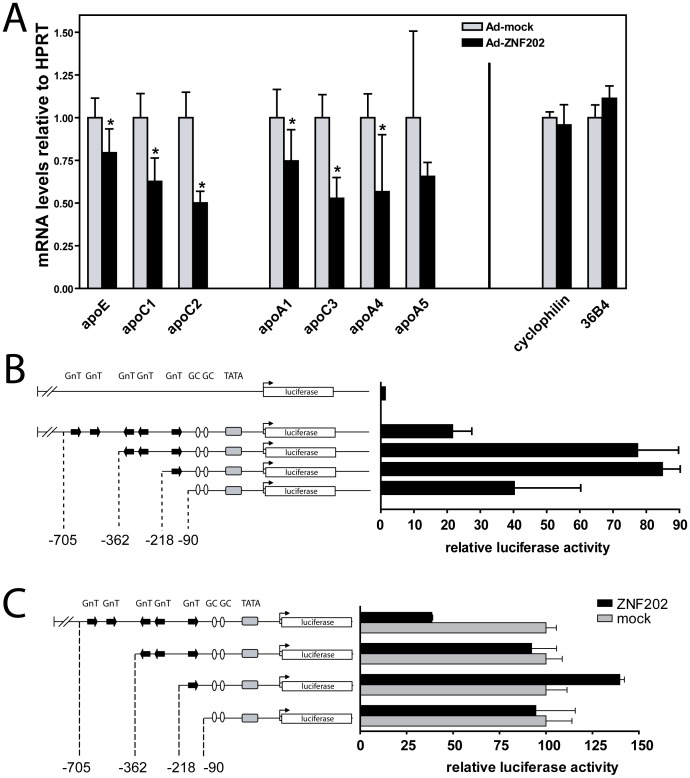
Znf202 overexpression leads to repression of members of the *apoe/c1/c2* and *apoa1/c3/a4/a5* gene clusters in mhAT3F2 and inhibits mouse apoE promoter activity. (A) MhAT3F2 cells were transduced with Ad.Znf202 (black bars) or Ad.LacZ (grey bars)(MOI = 100) and mRNA levels were measured via quantitative real time PCR at 24 hours post-infection. Data represent average of four transductions for each group (mean ± S.D) and expressions are relative to HPRT. (B) MhAT3F2 cells were transfected with mouse apoE promotor-reporter constructs, carrying a 729 bp fragment of the mouse apoE promoter lacking the downstream intron-1 (−705 to +24) or truncated variants thereof. Co-transfection with pCMV-LacZ served as a control for transfection efficiency. Luciferase activity was normalized for β-galactosidase activity (n = 4, mean ± S.D.). (C) MhAT3F2 cells were transiently cotransfected with the indicated reporter constructs and Znf202 expression vector (black bars). As control expression vector pShuttleCMV-empty was used (grey bars). After 24 hours cells were harvested and luciferase activities (n = 4, mean ± S.D.) measured and normalized for protein concentration.

The functionality of the generated recombinant plasmids and adenovirus carrying containing the *Znf202* gene construct was verified *in vitro*. As readout, we analyzed the effect of *Znf202* overexpression on the *Apoe/c1/c2* and *Apoa1/c3/a4/a5* gene clusters in mhAT3F2 cells. *Cyclophylin* or *36B4* expression by transduced mhAT3F2 cells was not affected by Ad.Znf202 transduction (Fig.1A). However, *Znf202* overexpression did result in a significant downregulation of apolipoprotein genes from both clusters. RNA levels were reduced up to 50% (ApoC2 and ApoC3) and reached significance for all genes (p<0.05) except *Apoa5*.

The repressive effect of Znf202 on the expression of one member of investigated apolipoprotein gene clusters, *Apoe*, has already been demonstrated and was therefore used to address the regulatory capacity of Znf202 [12], [18] and to identify its interaction with the mouse ApoE promoter sequence in a reporter assay. The presence of nucleotide sequences spanning −362 to −90 was seen to enhance promoter activity in mhAT3F2 cells as expected [25], [26], while the addition of region −705/−362 attenuated the enhanced activity to up to 4-fold (Fig.1B). To investigate the repressive effect of Znf202 on the ApoE promoter activity, we co-transfected various promoter-reporter constructs and Znf202 expression vector. Znf202 appeared to repress transcriptional activity of the ApoE promoter only when the complete −705/+24 region was present (Fig.1C). Deletion of the 5′ end (−705/−362) abolished the repressive effect of Znf202 on the ApoE promoter activity.

**Figure 2:**
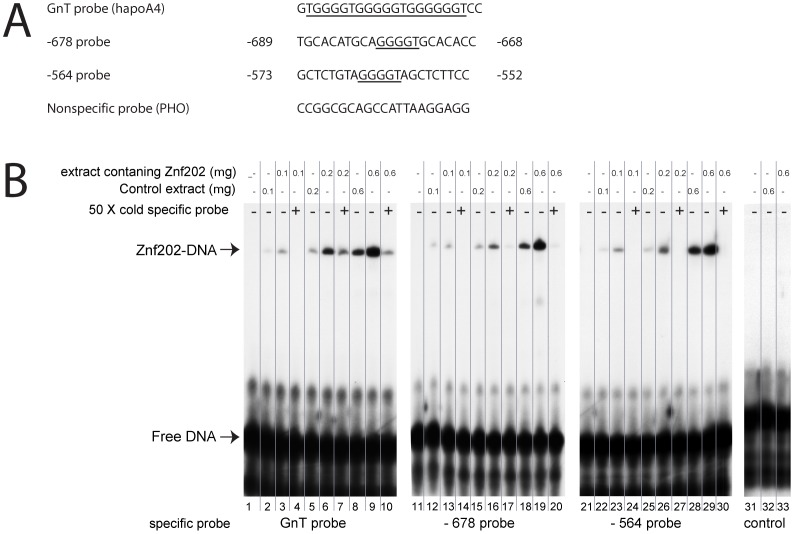
The Znf202 specifically binds to the alleged response elements −**678 and** −**564 within the** −**705/−362 region of the mouse apoE promoter.** (A) DNA fragments used in this study containing the putative Znf202 binding sequence. The putative consensus sequences are underlined. (B) EMSA with labeled DNA fragments GnT (lanes 1–10), −678 (lanes 11–20), −564 (lanes 21–30), and PHO (lanes 31–35) described above. Reactions contained the indicated amount (0.1–0.6 µg) of whole cell extract made from either control 911 cells, or from 911 cells overexpressing Znf202. Competition experiments using 50-fold excess of unlabeled GnT oligonucleotide (lanes 4,7 and 10), −678 oligonucleotide (lanes 14,17 and 20), or -564 oligonucleotide (lanes 24,27 and 30) confirmed the specificity of Znf202 binding to both GnT elements. Arrowheads indicate the mobility of unbound DNA and Znf202 protein bound DNA.

With the Znf202 repressive effect on ApoE promoter activity restricted to the −705/−362 region, we studied the interaction of Znf202 with the two putative GnT sites within this domain at positions −678 and −564 (Fig. 2A) by EMSA. The GnT control probe (Fig. 2B, lanes 1–10) induced a clear mobility shift indicative of the formation of DNA-protein complexes in extracts from Znf202 overexpressing 911 cells (lanes 3, 6, 9). Probe binding was specifically competed by 50-fold excess of unlabeled GnT probe (lane 4, 7, 10). Similar mobility shifts were observed for the [^32^P]-678 (lanes 11–20) or [^32^P] −564 probe (lanes 21–30) after Znf202 overexpression. In analogy to the reference probe, the specificity of binding was confirmed by displacement by a 50-fold excess of either unlabeled −678 or −564 probe. Finally, the extracts were unable to form DNA-protein complexes with an irrelevant PHO consensus probe, even at high concentrations (lanes 32 and 33). These in vitro results confirm the repressive role of Znf202 and establish the functionality of our murine Znf202 constructs.

### HDL Cholesterol Levels Markedly Reduced after Five Days of Hepatic Znf202 Overexpression

To address the role of Znf202 in lipid metabolism *in vivo,* we have overexpressed *Znf202* in two mouse strains: normolipidemic C57Bl/6J (WT) and hyperlipidemic *Ldlr−/−* by adenoviral gene transfer. Relative baseline expression of endogenous hepatic *Znf202* showed no difference between both mouse models (data not shown). The expression level of Znf202 is relatively low (C_T_ ∼ 30). Thus, the injection of Ad.Znf202 in *Ldlr−/−* and WT mice resulted in a substantial hepatic overexpression of the transgene after 5 days from ΔC_T_ = −4 (Ad.mock) to ΔC_T_ = 4 and 5, respectively (Fig. S1; relative expression compared to control genes, using real-time PCR analysis; P<0.001). Absolute expression levels of the housekeeping genes remained unaltered after Ad.Znf202 treatment.

**Figure 3:**
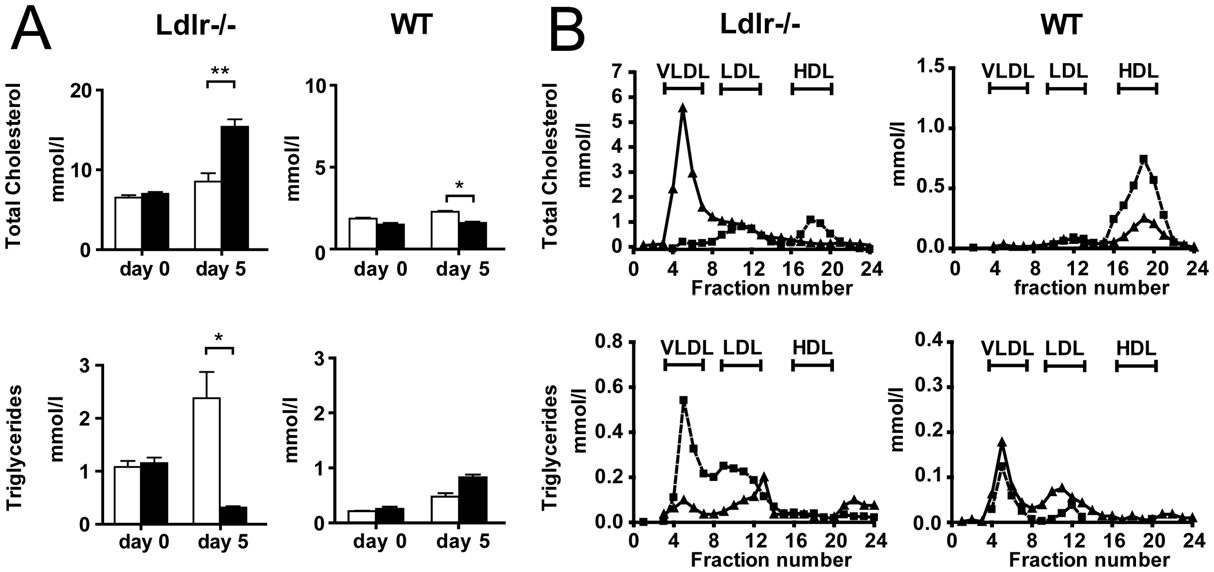
Hepatic Znf202 overexpression reduces HDL-cholesterol in *Ldlr−/−* and WT mice. Blood samples were drawn from *Ldlr−/−* (n = 4; left panels) and WT mice (n = 4; right panels) 5 days after injection with 2.10^9 ^pfu of Ad.Znf202 (filled bars) or with Ad-mock (open bars) and derived plasma was analyzed for triglyceride and total cholesterol content (A). Lipoprotein profiles were determined from *Ldlr−/−* (left panels) and WT mice (right panels) 5 days after injection with Ad.Znf202 (triangles) or with Ad-Mock (squares). The elution fractions were tested for triglyceride and total cholesterol content (B). * and ** indicates p<0.05 and p<0.001, respectively.

Plasma total cholesterol (TC) levels were 2-fold increased in Ad.Znf202 treated *Ldlr−/−* mice on day 5 after gene transfer (P<0.001), whereas in WT mice Znf202 overexpression had a slight lowering effect on plasma TC levels (Fig. 3A). Surprisingly, triglyceride (TG) levels were reduced by 4-fold (P<0.05) in *Ldlr−/−* mice only. The elevated plasma TC levels *in Ldlr−/−* mice overexpressing *Znf202* appeared to be attributable to an increased cholesterol content of the VLDL pool (Fig. 3B). In keeping with its presumed role in hypoalphalipoproteinemia, Znf202 overexpression led to a marked reduction in HDL-cholesterol both in WT and in *Ldlr−/−* mice (−63% and −70% respectively).

### Hepatic Znf202 Overexpression Induces Hepatosteatosis in Ldlr−/−

**Figure 4:**
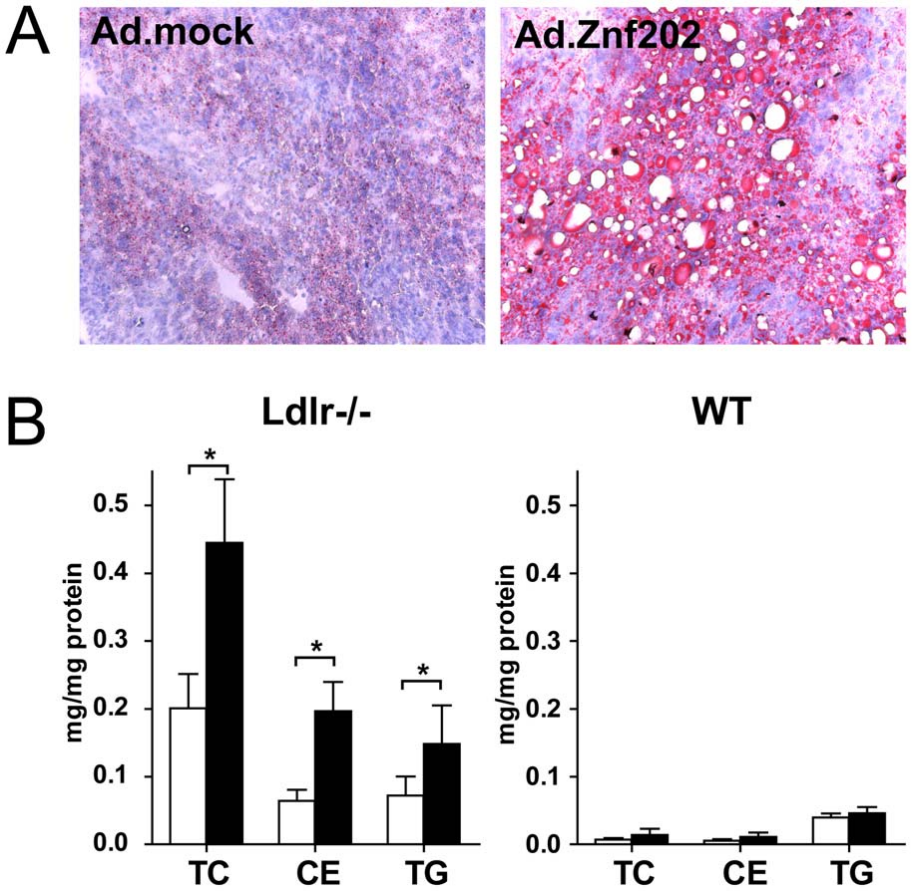
Hepatic Znf202 overexpression causes hepatosteatosis in *Ldlr−/−* mice only. Livers were isolated from *Ldlr−/−* and WT mice 5 days after injection with 2.10^9 ^pfu of Ad.Znf202 (filled bars) or Ad-mock (empty bars). Cryosections were prepared from Ldlr−/− liver samples and stained with Oil Red-O (A). Hepatic lipids were extracted from homogenized liver samples and cholesterol and TG concentrations were determined (B). Values are expressed as µg lipid per mg tissue protein and are means ± SD (n = 4). * indicates p<0.05.

Histological analysis of livers isolated from Ad.Znf202 and Ad.Mock treated *Ldlr−/−* and WT mice at day 5 post transduction revealed some striking differences. In *Ldlr−/−* mice, *Znf202* overexpressing livers were characterized by massive oil-red-O stained lipid deposition in intracellular vacuoles mainly (Fig. 4A), while liver morphology of *Znf202* transduced WT mice was normal (data not shown). As liver morphology of Ad.Znf202 treated mice was highly reminiscent of steatosis, we assayed the liver lipid content quantitatively. As can be appreciated from figure 4B, *Znf202* overexpressing livers from *Ldlr−/−* mice contained over 2-fold higher TC, cholesteryl esters (CE), and TG. In line with their normal liver morphology, no changes in hepatic lipid content were observed in *Znf202* transduced WT mice.

### Gene Expression Profile After Adenovirus Mediated Gene Transfer of *Znf202*

Since Znf202 has been reported to be a potent transcriptional repressor of various key genes in lipoprotein metabolism [12], we mapped the hepatic expression profile of a range of putative Znf202 responsive genes at day 5 after Ad.Znf202 or Ad.mock treatment of *Ldlr−/−* and WT mice. Unexpectedly, analysis of the *Apoe/c1/c2* and *Apoa1/c3/a4/a5* gene clusters showed that *Znf202* overexpression even increased the expression of *Apoc2* and *Apoa5* in WT (1,5-fold) and *Ldlr−/−* mice (3-fold) (both P<0.05) (table 1). In contrast, *Znf202* overexpression did not change *Apoe, Apoa1, Apoc1, and Apoc3* expression, even though these genes were seen to be Znf202 responsive in previous *in vitro* studies [5], [11]. Only *Apoa4* expression was found to be significantly reduced upon *Znf202* overexpression in WT mice.

**Table 1:** Relative gene expression in livers 5 days after infection with Ad-mock or Ad-Znf202 in Ldlr−/− and wild type mice.

	Ldlr−/−		Wild Type	
	Ad-mock	Ad-Znf202	Ad-mock	Ad-Znf202
ApoE	1.00±0.47	0.97±0.40	1.00±0.23	1.10±0.18
ApoC1	1.00±0.28	0.78±0.39	1.00±0.43	1.15±0.10
ApoC2	1.00±0.65	3.27±0.36*	1.00±0.28	1.61±0.21*
ApoA1	1.00±0.62	0.65±0.33	1.00±0.27	0.83±0.20
ApoC3	1.00±0.54	0.93±0.25	1.00±0.35	0.90±0.15
ApoA4	1.00±0.57	0.81±0.30	1.00±0.50	0.37±0.19*
ApoA5	1.00±0.35	2.87±0.31*	1.00±0.11	1.53±0.29*
Abca1	1.00±0.31	1.25±0.21	1.00±0.24	1.08±0.22
Abcg5	1.00±1.06	0.71±0.67	1.00±0.47	0.80±0.37
Abcg8	1.00±0.59	0.83±0.40	1.00±0.50	0.99±0.21
Ldlr	–	–	1.00±0.28	0,88±0,22
Sr-b1	1.00±0.98	0.51±0.39	1.00±0.27	0.91±0.29
Hmg-CoA red.	1.00±0.92	0.58±0.13	1.00±0.31	0.93±0.13
Cyp7a1	1.00±0.51	1.09±0.73	1.00±0.72	3.00±0.24*

Values are expressed as means ± SD.

* Indicates a significant difference (p<0.05) between Ad.Znf202 treated animals and their corresponding Ad.mock treated controls.

In search of a possible explanation for the elevated intrahepatic cholesterol levels, we determined gene expression levels of cholesterol related genes. *Znf202* overexpression did not alter gene expression of major cholesterol transporters *Abca1*, *Abcg5*, *Abcg8*, *Sr-b1,* and *Ldlr* (in WT). The gene expressions of *Abcg1* and LDL receptor related protein (*Lrp*) were unaffected as well (table S3). With regard to cholesterol synthesis, no change in the expression of 3-hydroxy-3-methyl-glutaryl-CoA reductase (*HMG-CoA reductase*) was observed. Interestingly, the bile acid synthesis gene cholesterol 7α-hydroxylase (*Cyp7A1*) was differentially affected between WT and *Ldlr−/−* mice upon *Znf202* expression. In *Ldlr−/−* it was unchanged while it was increased in WT (>3-fold). The increased cyp7A1 expression was confirmed at a protein level, as liver extracts from Ad.Znf202 treated WT but not *Ldlr−/−* mice showed sharply enhanced cyp7A1 activity (Fig. S2).

Lipid homeostasis is under tight control of nuclear receptors [27]. Next, we investigated whether Znf202 could have influenced apolipoprotein and lipid flux gene expression in an indirect manner by modulating nuclear receptor dependent regulatory pathways. The transcription factors *Hnf-4*, *Pparα*, *Pparδ*, *LXRα/β* and *FXR* were downregulated in *Ldlr−/−* mice by 25 to 60% (P<0.05) (table S3), while surprisingly no effect was found in WT mice.

As a measure of inflammatory responses potentially inflicted by adenoviral gene transfer we assessed hepatic MARCO and CD68 mRNA levels. The expression of both genes remained essentially unaltered by Znf202 treatment (1.0±0.8 and 1.0±.0.6 for Ad-mock versus 0.7±0.4 and 1.2±0.2 for Ad.Znf202 treated *Ldlr−/−* mice), thus confirming the histological finding that Znf202 did not promote massive leukocyte recruitment to the liver and excluding that adenovirus elicited inflammatory responses are underlying the observed phenotype in *Ldlr−/−* mice.

### Bile Flux Genes Repressed After 24 hrs of Hepatic Znf202 Overexpression in vivo

**Figure 5:**
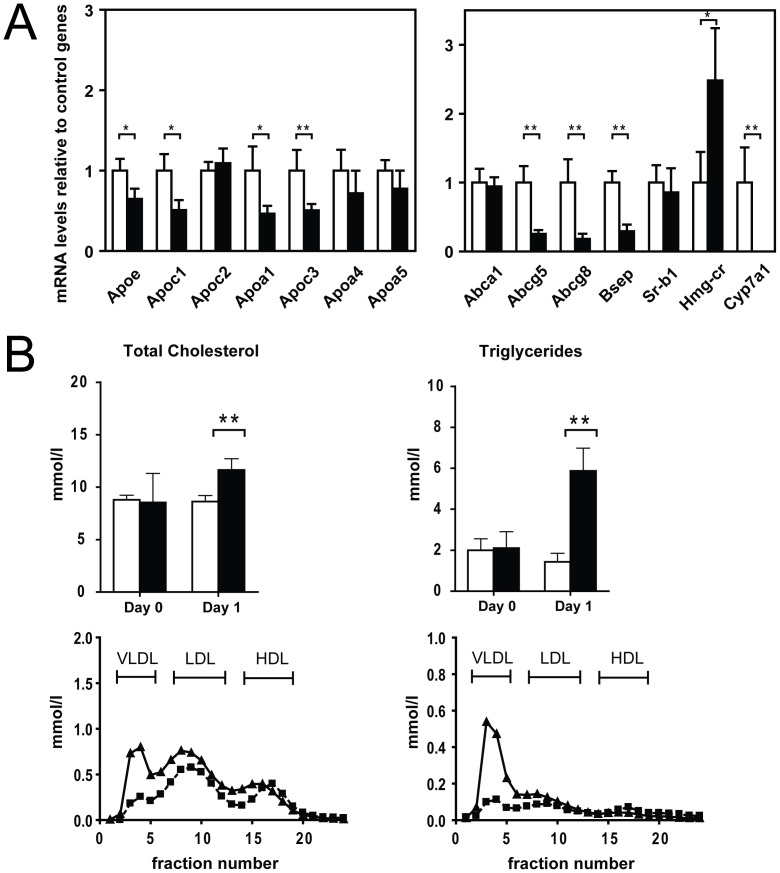
Znf202 overexression results in the reduced lipid related gene expression and affects plasma lipid levels *in vivo*. mRNA levels relative to control genes in livers from *Ldlr-* 24 hrs after injection with Ad.Znf202 (filled bars) or Ad.mock (empty bars) (A). Plasma samples were taken before (day 0) and 1 day after injection with Ad.Znf202-treated (filled bars) and Ad.mock-treated animals (open bars) to determine the total plasma cholesterol and triglycerides content. Plasma samples obtained from day 1 after injection with Ad.Znf202 (triangles) or with Ad.mock (squares) were also analyzed for lipoprotein profile (B). Data are means ± S.D (n = 5). * and ** indicates p<0.05 and p<0.001 respectively.

Despite hypoalphalipoproteinemia and the dramatic changes in lipid levels, especially in *Ldlr−/−*, we did not observe any of the expected, repressive effects of Znf202 on hepatic expression of target genes *in vivo* at 5 day post-transduction. In addition, several lipid related nuclear receptors were dysregulated in Znf202 overexpressing *Ldlr−/−* mice, although none had been reported to be Znf202 responsive. Conceivably, the initial and suppressive effects of Znf202 overexpression are overruled by secondary effects, which may underly the severe phenotype observed in *Ldlr−/−* mice. To address this notion, we have monitored hepatic gene expression patterns and lipid homeostasis in the mouse model that was mostly affected, the *Ldlr−/−,* at 24 hours after transduction. In line with the *in vitro* results, this increase in hepatic Znf202 gene expression (Fig. S1; from ΔC_T_ = −14 to ΔC_T_ = −11) resulted in the reduced expression of several genes within both apolipoprotein gene clusters in the liver (Fig 5A). The mRNA levels of *Apoa1*, *Apoc1*, *Apoc1*, and *Apoe* were approximately 2-fold reduced. Interestingly, we also observed a lowered expression (>70%) of the sterol transporter genes *Abcg5* and *Abcg8* and the bile salt efflux protein (*Bsep*). Most notably, the expression of the bile acid synthesis gene cholesterol 7α-hydroxylase (*Cyp7a1*) was more that 1000 fold reduced upon treatment with Ad.Znf202 (P<0.001). The attenuated expression of these bile flux genes suggests that hepatic overexpression of Znf202 affects the biliary secretion. The expression of several lipid related transcription factors such as *Fxr*, *Pparα*, and *Srepb1* was also reduced by hepatic Znf202 overexpression (table S4). However, a well described target gene of Znf202, *Abca1*, was unaffected. Additionally, the expression of HMG-CoA reductase, an important enzyme in cholesterol synthesis, was increased.

Together with the altered hepatic gene expression pattern, hepatic Znf202 overexpression had an effect on lipid metabolism already at 24 h post infection. Total cholesterol and triglycerides (TG) levels were markedly elevated (Fig. 5B). These changes in plasma lipids could mainly be ascribed to increased very low density lipoprotein (VLDL). The accumulation of intrahepatic lipids as manifested after 5 days of hepatic Z*nf202* overexpression, could not be detected at this early time point (data not shown).

## Discussion

Based on genetic and *in vitro* studies, the transcriptional repressor Znf202 has been proposed as a key regulator of lipid homeostasis and in particular of lipid efflux [28]. However, while its repressor function *in vitro* is well established, the precise role of Znf202 in lipid metabolism *in vivo* remains to be addressed. Despite these initial studies that link Znf202 to HDL, Stene and colleagues were unable to demonstrate a direct correlation between genetic variation within the *znf202* gene and HDL cholesterol levels [13]. However, at least one genetic variant was seen to be predictive of a high risk for cardiovascular diseases [29]. In this study we are the first to show, through a gain of function approach involving adenoviral *Znf202* gene transfer, a role of *Znf202* in lipid metabolism in mice.

To confirm the repressive effect of our *Znf202* constructs, we overexpressed murine *Znf202* in a mouse hepatoma cell line and showed reduction of expression of the majority of genes within both apolipoprotein gene clusters. The repressive nature of this transcription factor was further established in studies with the murine ApoE promoter. Screening of the proximal region of the murine *Apoe* gene for potential Znf202 binding sites revealed five GnT consensus sites (positions −678, −564, −338, −315, −160), of which the first two were shown to carry functional suppressor elements. Removal of these sites ablated Znf202 responsiveness. In addition, Znf202 was seen to specifically interact with the GnT sites within the responsive −705/−362 region, confirming the specific DNA binding characteristics of Znf202. Because the intron regulatory element 1 (IRE1), situated between exon 1 and exon 2 of the *Apoe* gene, also contains regulatory elements, we tested the effect of IRE1 [25]. Even though its mere presence reduced promoter activity 2-fold, the overall pattern of expressional regulation by Znf202 was not affected (data not shown).

To investigate the role of Znf202 in vivo, the effect of hepatic znf202 overexpression on lipid homeostasis was assessed in hyper- and normolipidemic mice 5 days after adenoviral *Znf202* gene transfer. In keeping with earlier genetic and in vitro studies hepatic Znf202 overexpression was accompanied by hypoalphalipoproteinemia both under normolipidemic (WT mice) and hyperlipidemic conditions (*Ldlr−/−* mice). Furthermore, it was seen to cause hypercholesterolemia and hypotriglyceridemia with concommittant steatosis in *Ldlr−/−* but not WT mice. Hepatic lipid accumulation was not accompanied by hepatic inflammation excluding that our findings reflect an inflammatory response to adenoviral infection. However, except for *apoa4* in WT, we did not observe overt repression of members of the *Apoe/c1/c2* and *Apoa1/c3/a4/a5* gene clusters in liver of both normo- and hyperlipidemic mice. Also the expression of other lipid related genes including the znf202 target genes *Abca1* and *Abcg1* [30] was not affected by hepatic *Znf202* overexpression.

Although these *in vivo* data clearly link znf202 activity to hypoalphalipoproteinemia, the apparent lack of correlation between the in vitro data of us and others and the hepatic gene expression profiles at 5 days after adenoviral *Znf202* administration was a surprise. This discrepancy might be caused by changes in lipid driven transcriptional factors like *Ppar*, *Lxr*, and *Fxr* in response to Znf202 induced dyslipidemia. For this reason, we analyzed the immediate response in the mouse model that was most affected, the *Ldlr−/−*. After only 24 hours of hepatic *Znf202* overexpression, we did observe lowered expression of members of both apolipoprotein gene clusters. Interestingly, several key genes involved in bile flux and most notably *Cyp7a1* were profoundly repressed by Znf202 as well. Closer analysis of the promoter sequence of murine *Cyp7a1,* expression revealed a putative Znf202 binding (GnT) site at position −862 bp. Surprisingly, this effect on *Cyp7A1* expression coincided with an increase in Hmg-CoA reductase expression suggestive of augmented *de novo* hepatic cholesterol synthesis. A similar compensatory response to decreased hepatic cholesterol input was seen in studies on the inhibition of sterol absorption by ezetimibe [31], [32]. Conceivably, one of the initial responses to Znf202 overexpression may involve the reduced hepatic cholesterol uptake. Nonetheless, the suppressive effect of Znf202 overexpression on bile flux genes such as *cyp7A1* and the concomitant *Hmg-CoA reductase* upregulation, may explain the strong hepatic lipid accumulation in *Ldlr−/−* as apparent at day 5 post infection [33], [34].

Znf202 overexpression led to changed expression of the transcriptional regulators of lipid metabolism *Fxr*, *Srepb1, and Pparα*. FXR, a negative regulator of *Cyp7a1* expression, was down- and not upregulated. Upregulation of Srebp1 and Pparα has been associated with lipogenesis and hepatic accumulation of lipids, respectively [35]. It remains to be determined whether and how the observed reduced expressions can contribute to the hepatosteatosis in Znf202-treated *Ldlr−/−*.

In response to hepatic *Znf202* overexpression in hyperlipidemic *Ldlr−/−* mice, both VLDL-cholesterol and VLDL-triglycerides were increased within 24 hours. Although potentially reflecting a direct effect of Znf202, the increased VLDL secretion could well be the result of increased hepatic lipids as described previously [36], [37]. While the increase in VLDL-cholesterol persisted for at least 4 days, triglyceride levels were sharply reduced in the same time span. Our gene expression analysis revealed increased expression of *Apoa5* and *Apoc2* at 5 days of *Znf202* overexpression both in *Ldlr−/−* and WT mice. Both ApoA5 and ApoC2 have been implicated in TG metabolism and in particular TG hydrolysis. Studies by Schaap et al. [38], and by Pennacchio et al. [39], already demonstrated a clear inverse relationship between serum ApoA5 and VLDL-TG levels. Similarly, ApoC2 functions as cofactor in LPL-mediated TG hydrolysis [40] and ApoC2 deficiency in humans is associated with severe hypertriglyceridemia [41]. In line with these findings, chronic *Znf202* overexpression in *Ldlr−/−* mice indeed sharply reduced serum triglyceride levels compared to Ad-mock treated controls.

**Figure 6:**
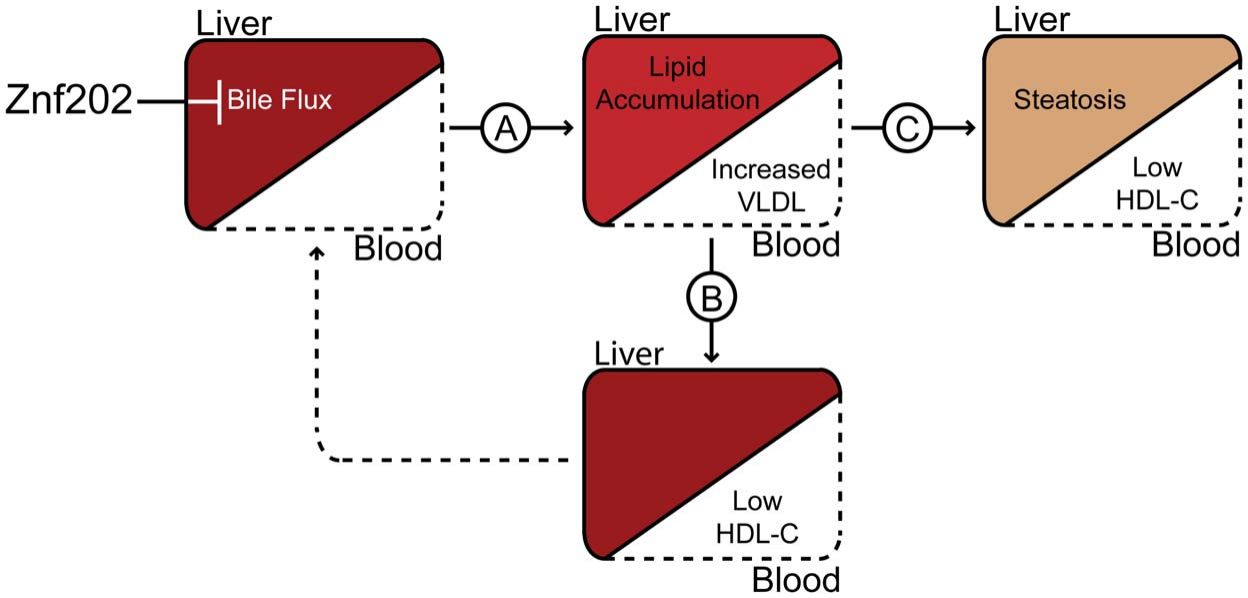
Proposed mechanism for Znf202. Elevated hepatic levels of Znf202 downregulate bile flux genes. Together with an increase in cholesterol synthesis and attenuated bile acid synthesis, this could result in the observed lipid accumulation in the liver and increased VLDL secretion (A). Under normolipidemic conditions, feedback mechanisms are able to reverse most of the initial effects of Znf202 overexpression (B). These initial effects cannot be sufficiently restored in mice under the hyperlipidemic conditions caused by the low density lipoprotein receptor deficiency. As a result, the mice become more hyperlipidemic and the lipid accumulation in the liver is followed by hepatic steatosis (C).

Whereas lipid homeostasis in hyperlipidemic LDLr−/− mice was strongly affected by *Znf202* overexpression, normolipidemic mice only showed mild effects. The moderate effect on total serum lipid levels in WT mice could be attributed in part to clearance via the LDLR. Moreover, additional secondary effects due to a progressively increased VLDL-TG lipolysis and panlobular lipid accumulation are not opportune in WT mice. In contrast to *Ldlr−/−*, the livers of WT mice show no accumulation of lipids upon Znf202 overexpression at day 5. Interestingly, while in *Ldlr−/−* the gene expression of *cyp7a1*, a key enzyme in bile acid synthesis [42], was apparently normalized after an initial strong Znf202 induced downregulation, it was strongly upregulated in normolipidemic mice. The latter response may underly the mild phenotype seen in WT mice which leads us to propose that the in vivo impact of Znf202 expression depends on the hyperlipidemic status (Fig. 6). Znf202 overexpression directly modulates bile flux gene expression resulting in hepatic lipid accumulation and a subsequent increase in VLDL secretion. Whereas initial effects on many of the established znf202 responsive hepatic genes appear to be compensated by a yet unknown mechanism, Znf202 overexpression did result in reduced HDL-cholesterol levels both in normo- and in hyperlipidemic mice. Whether or not *Znf202* is the responsible gene within the identified chromosomal region that has been linked to hypoalphalipoproteinemia in the study with Utah pedigrees [11] and has a direct or indirect association with the increased risk of coronary heart disease requires further investigation.

The relative levels of Znf202 mRNA in the livers were substantially high upon injection with Ad.Znf202, especially after 5 days (Fig. S1). Part of the explanation is probably the low endogenous hepatic expression of this regulatory factor measured in both mouse models before treatment. Therefore, the achieved relative mRNA levels after the gene transfer appear very high compared to the control group. Nonetheless, our finding corroborates previous reports that Znf202 is linked to HDL metabolism [11], [28]. Yet, how exactly Znf202 affects circulating HDL remains unclear. The initial reduction in expression of ApoA1, a major component of HDL, could partly explain the lower HDL levels observed at later time points after znf202 overexpression. In part, it might be associated with the induced apoA5 expression in both WT and *Ldlr−/−* mice. Previous studies have shown a significant and dose-dependent decrease in serum HDL levels after *Apoa5* overexpression in WT mice [43]. Of note, the expression of two other putative Znf202 targets that potentially can influence HDL levels, i.e. lipoprotein lipase and lecithin: cholesteryl acetyl transferase, were not affected in the liver [12], [44], [45] (tables S3 and S4).

In conclusion, we are the first to deliver *in vivo* proof for a functional role of Znf202 in lipid homeostasis and that znf202 overexpression causes hypoalphalipoproteinemia both in normolipidemic and hyperlipidemic mouse models. In mice lacking *Ldlr, Znf202* overexpression also was seen to induce severe hepatic lipid accumulation and dyslipidemia. These mice develop pronounced steatosis within 5 days, after initial downregulation of the expression of key genes in cholesterol and bile salt transport and bile acid formation. The *in vivo* results obtained with *Ldlr−/−* suggest that genetic defects in Znf202 might have even more impact in hyperlipidemic subjects. This study thus establishes *in vivo* a key role of Znf202 in HDL metabolism and in maintaining lipid homeostasis.

## Supporting Information

Figure S1 **Increased hepatic Znf202 mRNA levels after injection with Ad.Znf202 compared to Ad.mock.** Znf202 mRNA levels relative to control genes in livers were determined 24 hrs (*Ldlr−/−* mice; n = 5) and 5 days (WT and *Ldlr−/−* mice; n = 4) after injection with Ad.Znf202 (filled bars) or Ad-mock (empty bars). Data are means ± S.D.
(TIF)

Figure S2 **Liver analysis for Cyp7A1 activity revealed a significant Cyp7A1 induction in WT mice but not in** ***Ldlr−/−*** **mice at 5 days post-infection.** Liver was excised from *Ldlr−/−* and WT mice 5 days after injection with 2.10^9 ^pfu of Ad.Znf202 (filled bars) or Ad-mock (empty bars) and cyp7A1 activity was measured. Data are means ± S.D. of N = 4 determinations and ** indicates p<0.001.
(TIF)

Table S1 **Primers used to generate the apoE promotor constructs.**
(TIF)

Table S2 **Primers sets used for quantitative real-time PCR.**
(TIF)

Table S3 **Relative gene expression in livers 5 days after infection with Ad-mock or Ad-Znf202 in Ldlr−/− and wild type mice.** Values are expressed as means ± SD.
(DOC)

Table S4 **Relative gene expression in livers 24 hours after infection with Ad-mock or Ad-Znf202 in Ldlr−/−.** Values are expressed as means ± SD.
(DOC)

Text S1 **Supplementary material and methods.**
(DOC)
