# Supplementary figures and images for: Znf202 Affects High Density Lipoprotein Cholesterol Levels and Promotes Hepatosteatosis in Hyperlipidemic Mice
Source: PLoS One. 2013 Feb 28;8(2):e57492. doi: 10.1371/journal.pone.0057492 (PMC3585336; doi:10.1371/journal.pone.0057492)

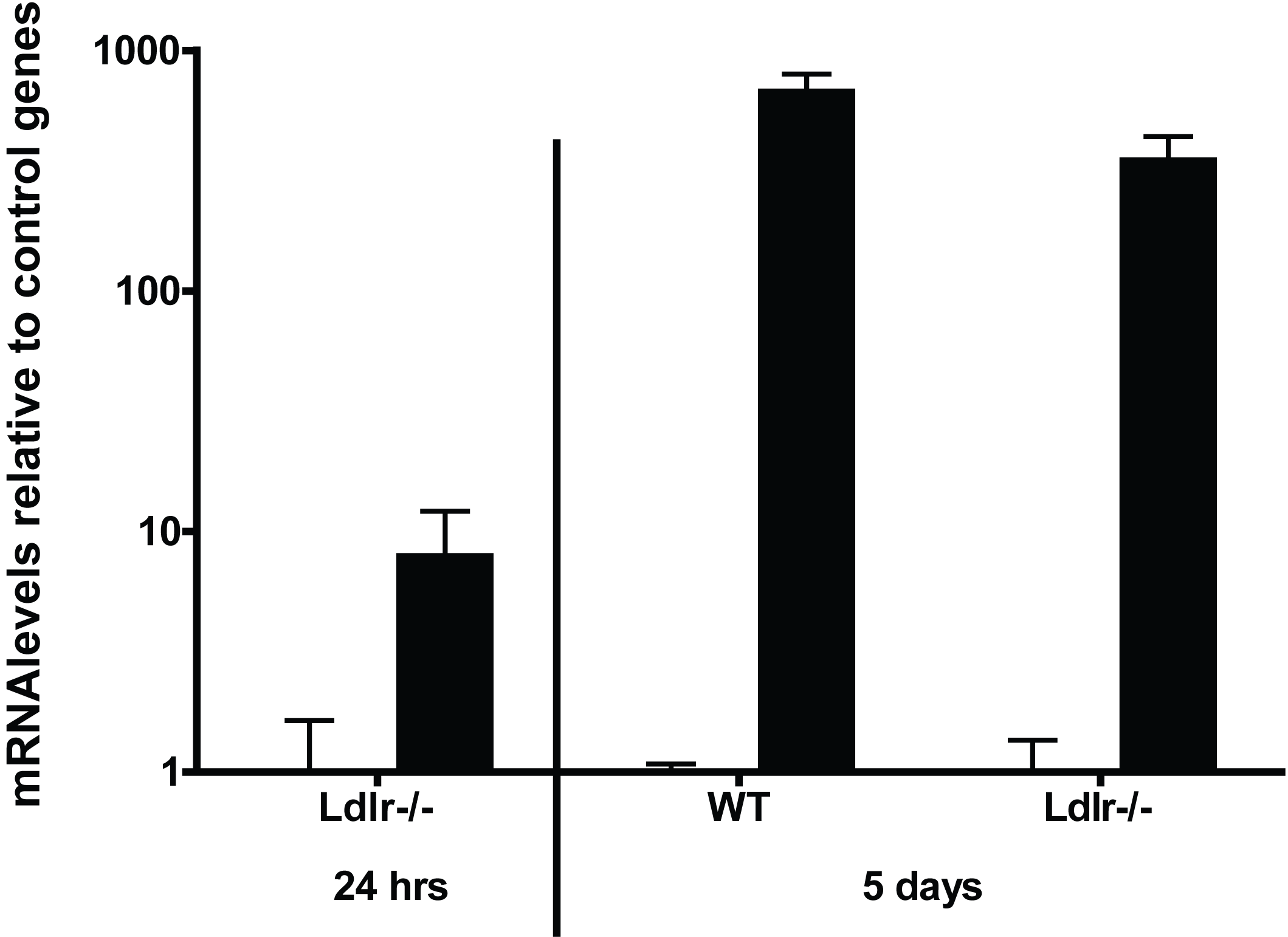

Supplement: Figure S1 — Increased hepatic Znf202 mRNA levels after injection with Ad.Znf202 compared to Ad.mock. Znf202 mRNA levels relative to control genes in livers were determined 24 hrs (Ldlr−/− mice; n = 5) and 5 days (WT and Ldlr−/− mice; n = 4) after injection with Ad.Znf202 (filled bars) or Ad-mock (empty bars). Data are means ± S.D. (TIF) [file pone.0057492.s001.tif]

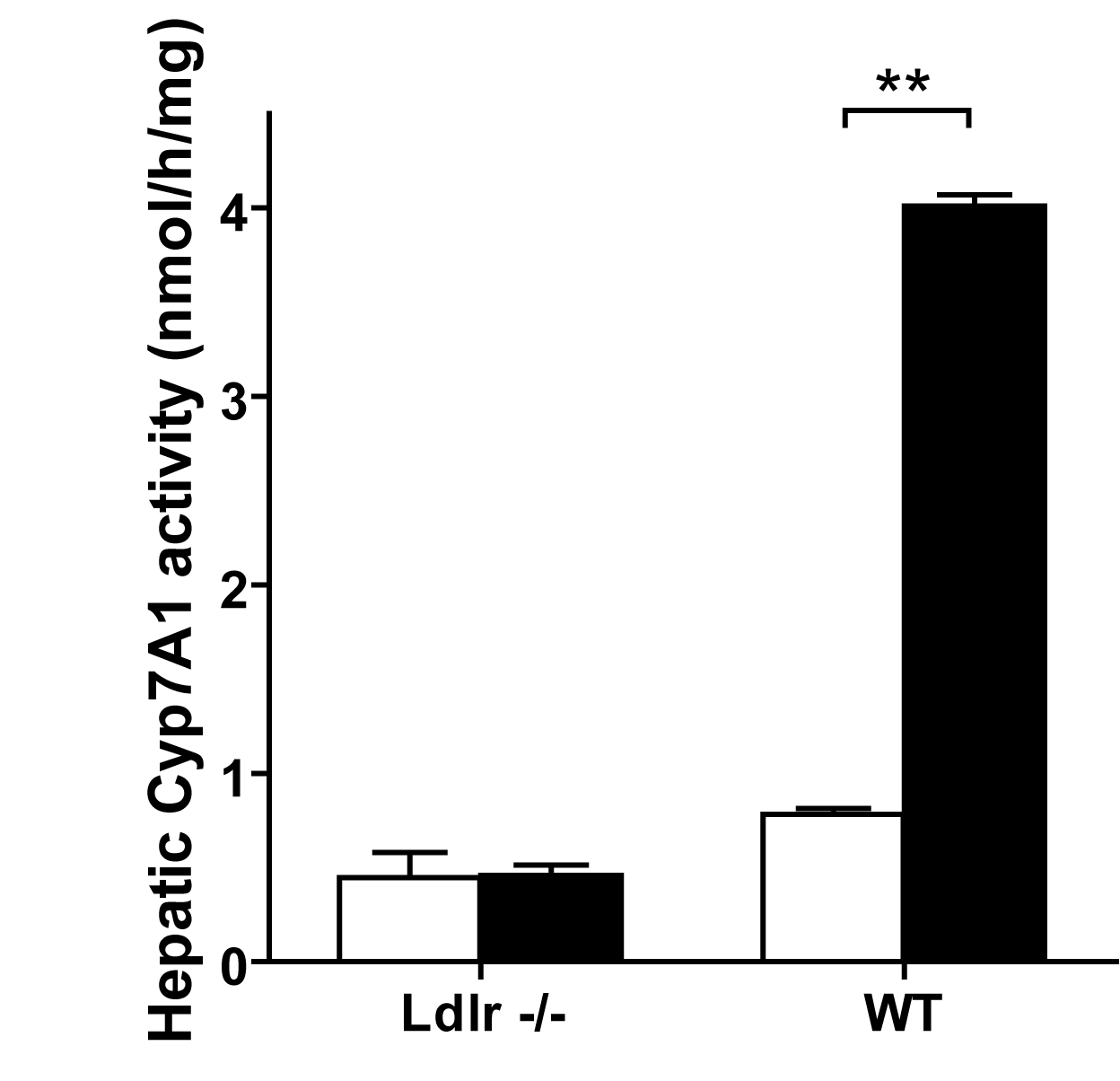

Supplement: Figure S2 — Liver analysis for Cyp7A1 activity revealed a significant Cyp7A1 induction in WT mice but not in Ldlr−/− mice at 5 days post-infection. Liver was excised from Ldlr−/− and WT mice 5 days after injection with 2.109 pfu of Ad.Znf202 (filled bars) or Ad-mock (empty bars) and cyp7A1 activity was measured. Data are means ± S.D. of N = 4 determinations and ** indicates p<0.001. (TIF) [file pone.0057492.s002.tif]

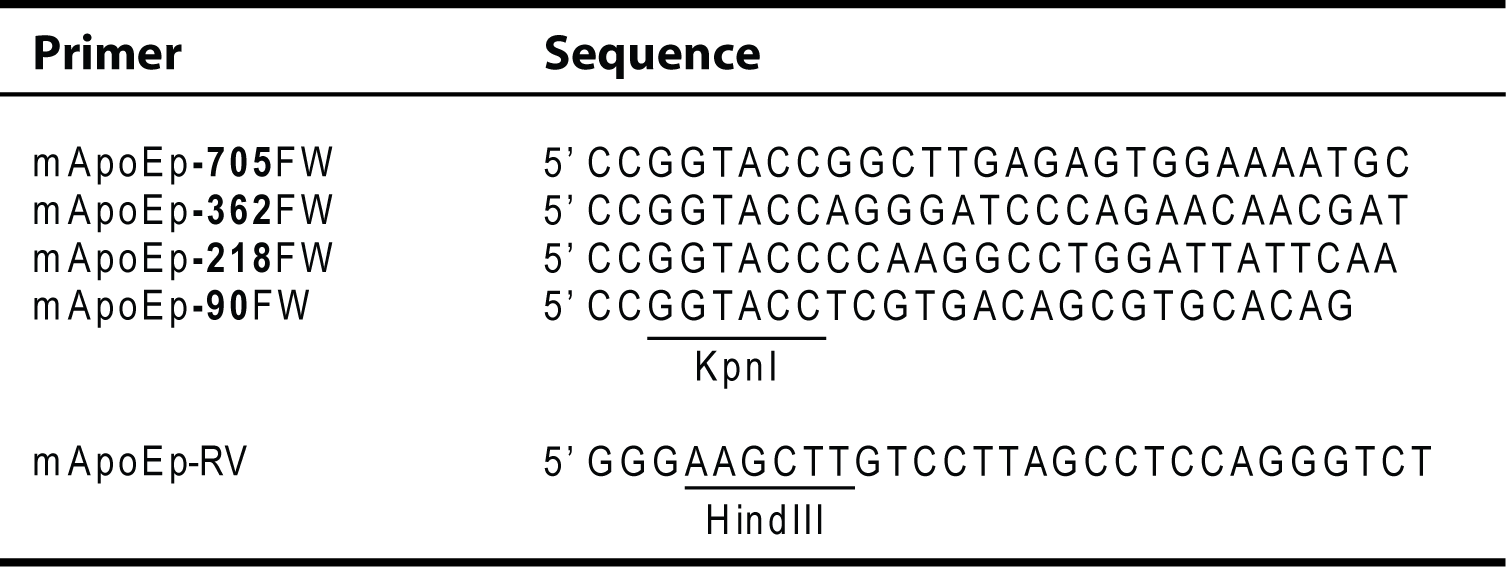

Supplement: Table S1 — Primers used to generate the apoE promotor constructs. (TIF) [file pone.0057492.s003.tif]

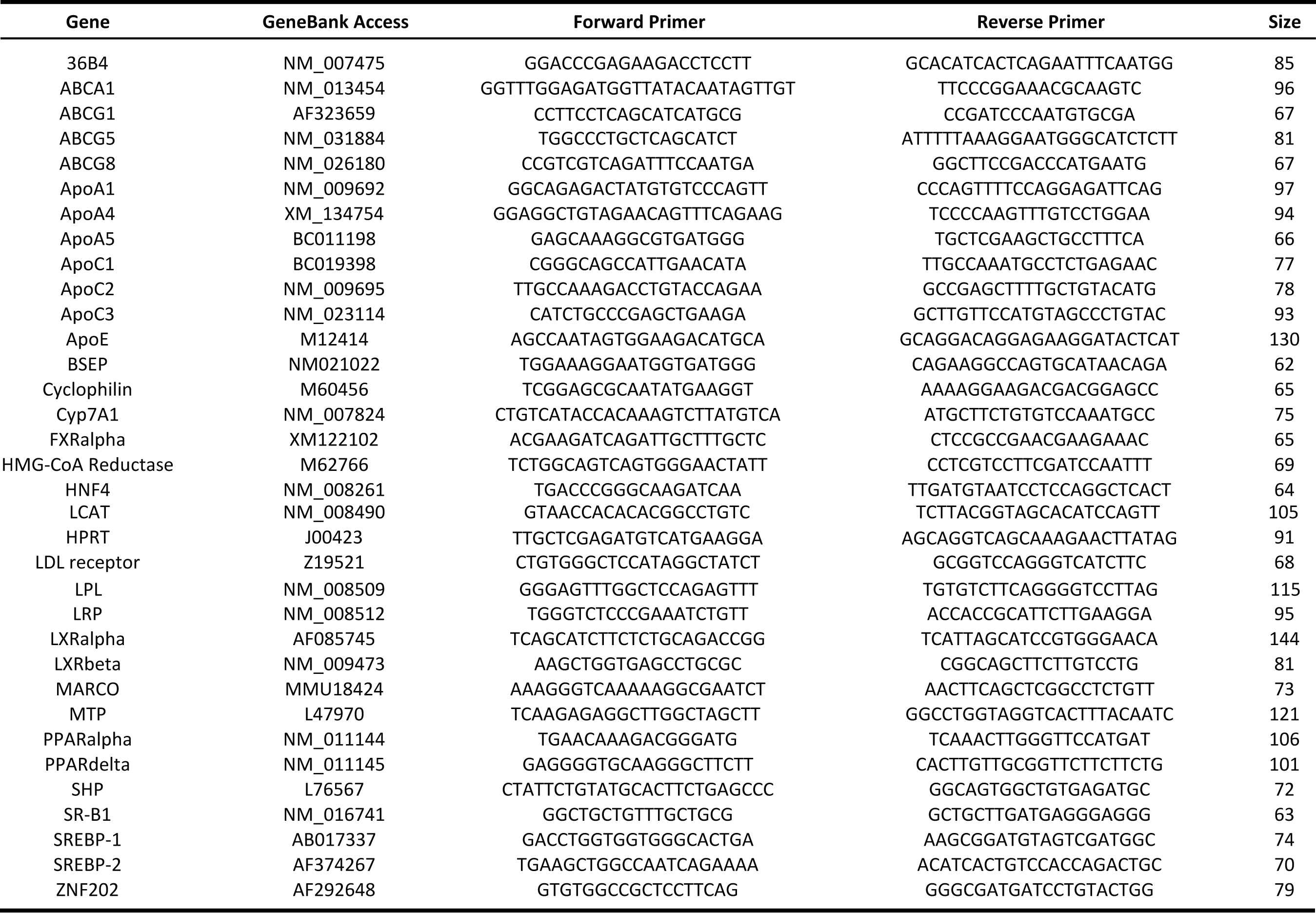

Supplement: Table S2 — Primers sets used for quantitative real-time PCR. (TIF) [file pone.0057492.s004.tif]
