# Supplementary material for: Znf202 Affects High Density Lipoprotein Cholesterol Levels and Promotes Hepatosteatosis in Hyperlipidemic Mice
Source: PLoS One. 2013 Feb 28;8(2):e57492. doi: 10.1371/journal.pone.0057492 (PMC3585336; doi:10.1371/journal.pone.0057492)
Supplement: Table S3 — Relative gene expression in livers 5 days after infection with Ad-mock or Ad-Znf202 in Ldlr−/− and wild type mice. Values are expressed as means ± SD. (DOC) [file pone.0057492.s005.doc]

**Table S3.**

|  | Ldlr-/- | | Wild Type | |
| --- | --- | --- | --- | --- |
|  | Ad-mock | Ad-Znf202 | Ad-mock | Ad-Znf202 |
| Abcg1 | 1.00 ± 0.30 | 1.18 ± 0.29 | 1.00 ± 0.62 | 1.66 ± 0.32 |
| Lrp | 1.00 ± 0.18 | 1.10 ± 0.46 | 1.00 ± 0.38 | 0.98 ± 0.14 |
| LXRα | 1.00 ± 0.26 | 0.53 ± 0.16* | 1.00 ± 0.39 | 0.86 ± 0.29 |
| LXRβ | 1.00 ± 0.83 | 0.53 ±0.18 | 1.00 ± 0.17 | 1.34 ± 0.30 |
| FXR | 1.00 ± 0.14 | 0.69 ± 0.09* | 1.00 ± 0.44 | 1.07 ± 0.24 |
| PPARα | 1.00 ± 0.21 | 0.55 ± 0.19* | 1.00 ± 0.46 | 1.10 ± 0.19 |
| PPARδ | 1.00 ± 0.41 | 0.43 ± 0.37* | 1.00 ± 0.38 | 0.73 ± 0.21 |
| HNF4 | 1.00 ± 0.31 | 0.49 ± 0.07* | 1.00 ± 0.36 | 0.69 ± 0.04 |
| Srebp1 | 1.00 ± 0.66 | 0.49 ± 0.29 | 1.00 ± 0.29 | 0.74 ± 0.17 |
| Srebp2 | 1.00 ± 0.80 | 0.42 ± 0.13 | 1.00 ± 0.34 | 0.78 ± 0.20 |
| LPL | 1.00 ± 0.37 | 1.10 ± 0.50 | 1.00 ± 0.25 | 0.92 ± 0.34 |
| LCAT | 1.00 ± 0.40 | 0.80 ± 0.24 | 1.00 ± 0.21 | 0.89 ± 0.31 |

* Indicated a significant difference (p<0.05) between Ad-Znf202 treated animals and their corresponding Ad-mock treated controls
