# Supplementary material for: Znf202 Affects High Density Lipoprotein Cholesterol Levels and Promotes Hepatosteatosis in Hyperlipidemic Mice
Source: PLoS One. 2013 Feb 28;8(2):e57492. doi: 10.1371/journal.pone.0057492 (PMC3585336; doi:10.1371/journal.pone.0057492)
Supplement: Table S4 — Relative gene expression in livers 24 hours after infection with Ad-mock or Ad-Znf202 in Ldlr−/−. Values are expressed as means ± SD. (DOC) [file pone.0057492.s006.doc]

**Table S4.**

|  | Ad-mock | Ad-Znf202 |
| --- | --- | --- |
| Lrp | 1.00 ± 0.29 | 0.50 ± 0.13* |
| Mtp | 1.00 ± 0.15 | 0.51 ± 0.08* |
| LXRα | 1.00 ± 0.16 | 0.74 ± 0.27 |
| LXRβ | 1.00 ± 0.18 | 0.83 ±0.31 |
| FXR | 1.00 ± 0.22 | 0.31 ± 0.19* |
| Shp | 1.00 ± 0.45 | 0.73 ± 0.64 |
| PPARα | 1.00 ± 0.09 | 0.25 ± 0.11* |
| PPARδ | 1.00 ± 0.14 | 1.05 ± 0.27 |
| HNF4 | 1.00 ± 0.42 | 0.62 ± 0.17 |
| Srebp1 | 1.00 ± 0.19 | 0.27 ± 0.04* |
| Srebp2 | 1.00 ± 0.26 | 1.28 ± 0.21 |
| LPL | 1.00 ± 0.75 | 1.37 ± 0.24 |
| LCAT | 1.00 ± 0.13 | 0.90 ± 0.24 |

* Indicates a significant difference (p<0.05) between Ad-Znf202 treated animals and their corresponding Ad-mock treated controls
