# Supplementary material for: Znf202 Affects High Density Lipoprotein Cholesterol Levels and Promotes Hepatosteatosis in Hyperlipidemic Mice
Source: PLoS One. 2013 Feb 28;8(2):e57492. doi: 10.1371/journal.pone.0057492 (PMC3585336; doi:10.1371/journal.pone.0057492)
Supplement: Text S1 — Supplementary material and methods. (DOC) [file pone.0057492.s007.doc]

**SUPPORTING INFORMATION**

**Supplementary Material & Methods**

**Whole cell extract preparation and electrophoretic mobility shift assays**

911 cells were transfected with 5 µg pAdTrackCMV-Znf202 or control plasmid on 10 cm dishes using LipofectAMINE plus kit. After 40 hours cells were harvested, and cell pellets were resuspended in 100 μl of a lysis buffer (20 mM Hepes, 600 mM KCl, 0.2 mM EDTA, 1mM DTT, 0.5 mM phenylmethylsulfonyl fluoride (PMSF), 0.5 mM leupeptine, 0.5 mM Aprotinin, 0.5 mM pepstatinA, 0.5 mM 4-(2-amino ethyl)benzene sulfonyl fluoride (ABSF)). Mixtures/suspension s were agitated gently for 30 minutes at 4ºC and centrifuged for 10 minutes at 4ºC. Supernatants were collected and an equal volume of a glycerol buffer was added (1 (20 mM Hepes, 40% glycerol, 0.2 mM EDTA, 1mM DTT, 0.5 mM phenylmethylsulfonyl fluoride (PMSF), 0.5 mM leupeptine, 0.5 mM Aprotinin, 0.5 mM pepstatinA, 0.5 mM 4-(2-amino ethyl)benzene sulfonyl fluoride (ABSF)). Protein concentrations were determined by a Bradford assay (BioRad). The DNA-binding activity of mouse Znf202 in whole cell extracts was studied by means of electrophoretic mobility shift assay (EMSA). Double-stranded DNA probes containing the Znf202 binding sites, the consensus GnT oligonucleotide, the putative -564 and -678 Znf202 binding oligonucleotides, as well as a control unrelated fragment containing the pleiohomeotic (PHO) consensus binding site were used for gel shift analyses after end-labeling of each probe with [
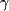
-^32^P]ATP and T_4_ polynucleotide kinase. The sequences of the GnT, -564, -678, and PHO oligonucleotides were 5'- GTGGGGTGGGGGTGGGGGGTCC-3', 5'- GCTCTGTAGGGGTAGCTCTTCC -3', 5'- TGCACATGCAGGGGTGCACACC -3' and 5'- CCGGCGCAGCCATTAAGGAGG-3', respectively. ^32^P labeled oligonucleotide probes (
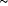
20 fmol) were incubated with 0.1 – 0.6 µg of whole cell extract of control or Znf202 overexpressing cells for 30 min at room temperature in 25 µl of reaction mixture containing 20 mM HEPES–KOH pH 7.9, 5 mM MgCl_2_, 75 mM KCl, 1 mM DTT, 0.1 mM EDTA, 2 µg of BSA and 2 µg of poly(dI–dC)· poly(dI–dC) as competitor DNA. Specificity of binding was determined by competition experiments in the presence of a 50-fold excess of unlabeled GnT, -564, or -678 oligonucleotide. Reaction mixtures were loaded on native 6% polyacrylamide gels, run in 0.5x Tris-Glycine at 200 V at 4°C, and visualized by autoradiography.

**Generation of Adenoviral Constructs**

Recombinant adenoviral plasmids by homologous recombination of pShuttleCMV-Znf202 with pAdEasy1 were generated in BJ5183 cells as described by He et al. [1]. Correct clones were propagated in STBL2 cells (Invitrogen Life Technologies, Breda, The Netherlands). For the production of Ad.Znf202 Per.C6 cells (Crucell, Leiden, NL) were transfected with 3 µg Pac-I-linearized adenoviral construct on 35 mm dishes using LipofectAMINE PLUS kit according to the manufacturer’s protocol (Life Technologies). After 16 hours transfection medium was replaced by growth medium. Transfected cells were harvested at day 7 post-transfection and after three freeze-thaw cycles the lysate was used for large-scale production of Ad.Znf202 in Per.C6 cells. Virus was purified by CsCl centrifugation steps and dialysis. Final yields were assessed via a plaque assay in 911 cells and titers were obtained of approximately 2 × 10^10^ plaque forming units (pfu)/ml. The construction of control virus Ad.mock (Ad-LacZ) has been described previously [2].

**Cholesterol 7α-hydroxylase enzyme activity assay**

Cyp7A1 enzyme activities in isolated liver microsomes were determined essentially according to Chiang [3] by measuring the conversion of cholesterol into 7
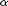
-hydroxycholesterol. In short, liver homogenate (750 ug protein) was incubated in 1 ml of buffer containing 0.1 M potassium phosphate, pH 7.2, 50 mM NaF, 5 mM DTT, 1 mM EDTA, 20% glycerol (w/v), and 0.015% (w/w) CHAPS. Twenty µl of 1 mg cholesterol in 45% (w/v) hydroxypropyl-ß-cyclodextrin was added and the mixture was incubated for 10 min at 37°C under gentle agitation. Regenerating buffer (200 µl) was added, containing 10 mM sodium isocitrate, 10 mM MgCl_2_, 1 mM NADPH, and 0.15 U isocitrate-dehydrogenase at 37 °C. After 20 min of incubation, a stop solution (60 µl) containing 20% sodium cholate and 1 µg 20
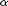
-hydroxycholesterol, which served as an internal standard, was added. Steroid products were oxidized for 45 min in 100 µl buffer containing 0.1% cholesterol oxidase, 10 mM K_2_HPO_4_, 1 mM DTT, and 20% glycerin at 37 °C, and the reaction was stopped by addition of 2 ml ethanol. Cholesterol metabolites from this reaction mixture were extracted in petroleum ether and the ether layer was evaporated under a stream of nitrogen. Residues were resuspended in a mixture of 60% acetonitril, 30% methanol and 10% chloroform (v/v) and were analyzed by using C-18 reverse phase HPLC on a Tosohaas TSK gel-ODS 80TM column equilibrated with 70% acetonitrile and 30% methanol at a flow rate of 0.8 ml/min. The amount of products formed was determined by monitoring the absorbance at 240 nm. Peaks were integrated using Data Control software (Cecil Instruments, UK).

Reference List

1. He TC, Zhou S, da Costa LT, Yu J, Kinzler KW, et al. (1998) A simplified system for generating recombinant adenoviruses. Proc Natl Acad Sci U S A 95: 2509-2514.

2. Kobayashi K, Oka K, Forte T, Ishida B, Teng B, et al. (1996) Reversal of hypercholesterolemia in low density lipoprotein receptor knockout mice by adenovirus-mediated gene transfer of the very low density lipoprotein receptor. J Biol Chem 271: 6852-6860.

3. Chiang JY (1991) Reversed-phase high-performance liquid chromatography assay of cholesterol 7 alpha-hydroxylase. Methods Enzymol 206: 483-491.
